# Socioeconomic Status Disparities in Children’s Cognition—Differences in Degree or Kind?

**DOI:** 10.1177/17456916251409785

**Published:** 2026-02-10

**Authors:** Lingyan Hu, Martha J. Farah

**Affiliations:** 1 Graduate School of Education, University of Pennsylvania; 2 Department of Psychology, University of Pennsylvania

**Keywords:** socioeconomic status, neurocognitive development, moderation analysis, diverse developmental pathways, differences versus deficits

## Abstract

The “socioeconomic achievement gap” refers to socioeconomic disparities in children’s academic outcomes. Do these gaps invariably reflect cognitive processes that are similar in kind across the socioeconomic status (SES) spectrum but differ quantitatively in their efficacy? Or, in some cases, do they reflect cognitive processes that differ, in kind, between higher and lower SES, that is, qualitatively? In this systematic review, we used the ways in which brain structure and function relate to cognitive performance to answer these questions, focusing on academically relevant cognitive abilities. Specifically, the brain correlates of performance served as a signal regarding the underlying cognitive processes used to perform cognitive tasks. The literature was searched for studies that reported whether SES moderated the brain–cognition relation. In 15 cases, significant moderation was found, suggesting that children from diverse SES backgrounds may use underlying brain systems differently to achieve cognitive task performance. Three general mechanisms are reviewed, as are the broader implications of qualitative differences for teaching and for the causal relations leading to socioeconomic disparities in cognition.

It is well documented that children’s cognitive ability and academic achievement are associated with their socioeconomic status (SES). For example, IQ scores, executive function (EF), and language abilities are positively related to SES (Falk et al., 2021; Lawson et al., 2018; Pace et al., 2017). Academic achievement, including reading and mathematics skills, is also higher in children from higher SES families (Reardon et al., 2014; Von Stumm, 2017) and have been related to SES disparities in cognitive abilities such as language (Lurie et al., 2021) and EF (Lawson & Farah, 2017). Understanding these gaps would make it possible to address them more effectively.

SES-related cognitive variation could result from *quantitative differences*, in which individuals from higher and lower SES backgrounds engage the same underlying neurocognitive processes but differ in the degree of efficiency or effectiveness of those processes. Substantial research has demonstrated quantitative disparities. Specifically, such research typically involves using mediation analyses to determine whether the SES relation to cognitive performance can be accounted for by neural differences. If SES–performance relations are mediated by neural differences, this suggests that children of higher SES perform better than their lower SES counterparts because they possess more of the needed neural resources.

For example, structural measures—both global (e.g., gray matter volume, white matter volume, total cortical surface area) and regional (e.g., volume of smaller regions of interest such as gyri or subcortical structures)—have been reported to mediate associations between SES and outcomes such as IQ (McDermott et al., 2019), academic achievement (Hair et al., 2015), EF (Noble et al., 2015), and vocabulary (Romeo et al., 2018). Evidence also comes from functional measures: Brain activation during cognitive tasks (e.g., working memory) mediates the SES–math achievement link (Finn et al., 2017), and resting-state functional connectivity patterns mediate SES differences in general cognitive ability (Rakesh & Whittle, 2021; Sripada et al., 2021).

Alternatively, individuals from different SES backgrounds may accomplish some tasks using their neurocognitive systems differently. Here, these differences are termed *qualitative*. In statistical terms, SES may moderate brain–cognition relations, either by interacting statistically with brain measures and performance or (if performance is equivalent at different levels of SES) by interacting statistically with brain measures and task conditions that isolate the cognitive process of interest. Both statistical forms—(a) an SES × Brain interaction predicting performance and (b) an SES × Condition interaction predicting neural responses under equivalent performance—provide evidence of qualitatively different neural mechanisms underlying task performance. In both cases, individuals at different points along the SES gradient may achieve similar behavioral outcomes through distinct patterns of brain activation. The former reflects SES moderating how brain activity relates to performance, whereas the latter reflects SES moderating how the brain responds to task demands when behavior is equivalent.

Recent years have seen growing attention to such qualitative differences (e.g., Demir et al., 2015). Yet the field still lacks a systematic review of empirical evidence supporting the existence of qualitative differences. The current article was undertaken because the effort to understand and reduce SES disparities depends on an accurate understanding of the nature of these disparities.

Evidence of qualitative differences is relevant to the long-standing distinction between “differences” and “deficits” in psychology and education (Williams, 1970). This distinction has been drawn to suggest that a mismatch between the ways in which lower SES children develop adaptive skills in their early environments and the ways in which they are taught and evaluated in educational and psychological contexts contributes to SES-related performance disparities. It also fits with the more recent idea that cognitive disparities between lower and higher SES children may be adaptations to their different environments (D’Angiulli, Lipina, & Olesinska, 2012; DeJoseph et al., 2024; Ellwood-Lowe et al., 2021; Frankenhuis et al., 2020; Noble et al., 2021; Taylor et al., 2023). We return to these interpretations of cognitive disparities and their relation to the quantitative–qualitative distinction in the Discussion section.

Here, the neural correlates of cognition are used to distinguish qualitative and quantitative SES differences in cognition. Two criteria are proposed in the selection of evidence. First, one needs a statistically significant interaction involving SES, the brain, and cognition. For the purposes of this analysis, cognition may be captured by cognitive performance or, for the few cases in which performance does not differ by SES, the conditions whose contrast isolates the cognitive process of interest. This indicates that the relation of the brain to cognition is not just descriptively different at different SES levels but is statistically significantly different. Second, one needs to rule out an artifactual cause of interaction effects, specifically ceiling or floor effects in the dependent measure of cognitive performance. If one SES group performs at or near the highest or lowest score possible, whereas the other group does not, then the first group will show attenuated effects of brain measures for the simple reason that the measurement scale has little or no room to show an effect of the brain measure on performance. The result will be a difference in the size of the brain measure effect for higher and lower SES participants, which may be statistically significant.

A few qualifying reports were initially identified that prompted a systematic search for additional studies, the results of which are presented in this article. Search criteria included participant age (children and adolescents) and cognitive domain (academically relevant domains of mathematics, language, reasoning, selective attention, and EF), as well as statistical analyses reported (a relevant statistical test of moderation) and data distributions (a range of SES levels and a range of behavioral performance and equivalent or overlapping performance between lower and higher SES, the latter of which was not attributable to ceiling or floor effects).

It should be noted that this search strategy cannot determine how much SES moderates brain–cognition relations because there is no way to know how many studies not reporting moderation simply never tested for it, or tested for it and deemed it either a uninteresting or uninterpretable (positive or negative) result. The goal of this article was therefore more modest but still meaningful: establishing the existence of moderation and hence of qualitative differences in the neural systems recruited for cognitive task performance at lower and higher SES levels.

## Method

### Literature search

The literature search was conducted on PubMed up to April 1, 2023, and adhered to the PRISMA guidelines. The search focused on identifying peer-reviewed empirical studies written in English that reported the presence or absence of SES moderation of brain–cognition relations. Medical Subject Headings (MeSH) was used to search relevant articles. MeSH is a controlled specialized thesaurus managed by the National Library of Medicine for indexing journal articles on PubMed. As subject descriptors, MeSH terms have the advantage of identifying studies on a similar topic despite variations in terminologies, facilitating a more efficient and comprehensive search (Dhammi & Kumar, 2014; Shultz, 2007).

The MeSH terms for the search string were constructed by looking up terms in National Library of Medicine’s MeSH browser and identifying those covering the key concepts pertinent to our review. “Qualifiers” were applied to some of the MeSH terms to refine the search to relevant research fields (formatted as “MeSHterm/Qualifier”). Two non-MeSH search terms, “NOT (patient)” and “NOT (country),” were also incorporated as an initial step to exclude articles focusing on populations with clinical disorders or on the poverty status of an entire country. The exact search string used to retrieve journal articles on PubMed was as follows:

((“socioeconomic factors”[MeSH Terms]) OR (“social class”[MeSH Terms]) OR (poverty/economics[MeSH Terms]) OR (poverty/psychology[MeSH Terms]) OR (“Educational Status”[MeSH Terms]) AND ((Brain[MeSH Terms]) OR (“Magnetic Resonance Imaging”[MeSH Terms]) OR (Electroencephalography[MeSH Terms]) OR (“Neuropsychological Tests”[MeSH Terms]) OR (“Diffusion Tensor Imaging”[MeSH Terms]) OR (Neuroimaging[MeSH Terms) OR (Evoked Potentials[MeSH Terms])) AND ((“language development”[MeSH Terms]) OR (reading[MeSH Terms]) OR (“Executive Function”[MeSH Terms]) OR (Mathematics[MeSH Terms]) OR (Academic Performance[MeSH Terms]) OR (Language[MeSH Terms]) OR (Cognition[MeSH Terms]) OR (Attention[MeSH Terms]) OR (Learning[MeSH Terms])) AND ((child[MeSH Terms]) OR (adolescent[MeSH Terms])) NOT (patient) NOT (country)

**Fig. 1.**
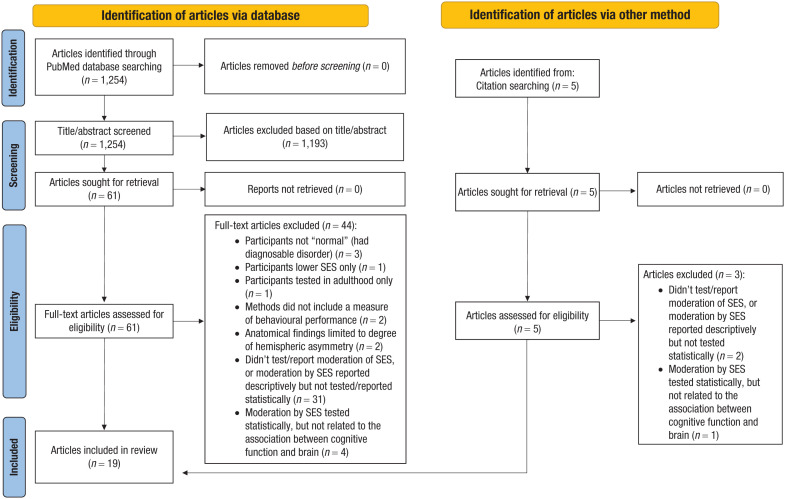
PRISMA flowchart of the study selection.

The MeSH search yielded a total of 1,254 results. The reference lists of selected articles and relevant review articles were also reviewed to identify potential articles to retrieve (for details, see Fig. 1).

### Inclusion and exclusion criteria

The first author (L. Hu) performed the searches and initially screened the resulting articles on the basis of the title and abstract for relevance, excluding nonempirical articles (e.g., reviews, meta-analyses), articles with special subject populations defined by clinical disorders or bilingualism, or articles lacking any mention of SES or brain measures in the abstract. Each article not excluded during this initial screening underwent further evaluation by both authors (L. Hu and M. J. Farah). To be included in the current article, articles needed to:

1. Have a sample that represented a range of SES backgrounds so there was enough variance in the SES measure, operationalized as either a dichotomous or continuous variable, to enable the statistical testing of SES effects. Articles focusing on only higher or lower SES populations were excluded.
2. Have at least one neural measure. Articles using only behavioral neuropsychological tests were excluded.
3. Have at least one measure of cognitive ability or performance to be analyzed in relation to the brain variable(s) or to establish that neural differences were not attributable to lower behavioral performance, which would suggest merely less effective deployment of the same neurocognitive processes. Articles without any cognitive behavioral measure were excluded.
4. Report a statistical analysis testing either (a) an interaction among SES, brain, and behavioral measures, or (b) an interaction between SES and task condition predicting neural responses when behavioral performance was equivalent across SES groups. For either form of interaction, the results must have been reported regardless of the significance of results. Articles that did not include such interaction terms (i.e., between SES and performance, SES and neural measures, or SES and condition) were excluded.
5. Not show floor or ceiling effects by the criterion of means lying within 1 *SD* of the maximum or minimum value based on graphs or descriptive statistics because this could artifactually create an interaction.
6. Have participants who were typically developing children or adolescents at the time of testing. Articles using adult participants reporting childhood SES or those using a sample of participants with developmental disorders were excluded.

Last, articles focusing on SES and brain lateralization were further excluded. Although differential involvement of the two hemispheres could be considered a kind of qualitative difference, homologous regions of the left and right hemispheres do not necessarily perform different cognitive functions. They may differ from one another qualitatively or quantitatively. This is particularly relevant to SES because lower SES has been associated with reduced lateralization (i.e., less asymmetry between left and right hemisphere) in both behavioral (Boles, 2011) and neuroimaging studies (Poeppl et al., 2022). Details about exclusion can be found in Figure 1.

### Unit of analysis

Articles identified by the method described above may have reported a single study and a single analysis, providing evidence (either positive evidence of moderation or a null result) where moderation is concerned. In these cases, an entire article would be cited as a single piece of evidence. Alternatively, it is also possible that single articles could have presented multiple studies or analyses, possibly pertaining to distinct cognitive domains. In these instances, the “unit of analysis” for evidence concerning moderation would be the individual study or analysis. Accordingly, our overview summarizes significant or null findings for each of these analyses separately according to the relevant cognitive domain: For example, studies investigating SES moderation in relation to both memory and the hippocampus and language and the hippocampus are referenced twice in the Results section, once in the Language subsection and again in the Memory subsection.

## Results

The search identified 19 relevant articles. Fifteen of these articles reported on the presence or absence of moderation by SES in a single cognitive task, and 11 of these 15 articles found evidence of moderation. The remaining four single-task studies reported no significant moderation. The four articles reporting on multiple cognitive abilities reported significant findings for some, but not all, of the abilities examined.

In the summary of results that follows, findings are grouped by six broad cognitive domains: mathematics, language, reasoning, selective attention, EF, and memory. For each cognitive domain, we report both significant and nonsignificant findings concerning moderation analysis. Table 1 summarizes the findings.

**Table 1.** Summary of Studies Organized by Proposed Mechanism of Qualitative SES Difference

Study	Participants	SES measures	Target cognitive functions	Neural measure	Main findings	Proposed interpretation and comments
Brito et al. (2017)	*N* = 1,091; age: 3–20 years	Family income and parental education (continuous)	(a) Vocabulary, oral reading; (b) inhibitory control, cognitive flexibility	Structural MRI	In children with thinner cortices, SES attenuated the negative association between cortical thickness and language; in children with thicker cortices, SES attenuated relations with inhibitory control and cognitive flexibility	Buffering
Conant et al. (2017)	*N* = 32; age: 7–12 years	Maternal education (continuous)	Phonemic discrimination	fMRI	Among children with low categorical discrimination ability, higher SES enabled the recruitment of the left MFG, IFG, SFG, and insula	Buffering
Leonard et al. (2019)	Children: *N* = 115 (age: 4–7 years); adolescents: *N* = 59 (age: 12–16 years)	Maternal education and income (continuous)	Matrix reasoning	Structural MRI	SES moderated Matrix Reasoning × Cortical Thickness associations in the bilateral RMFG—positive in lower SES and nonsignificant in higher SES (children only)	Buffering (what the authors referred to as “adaptation”)
Maguire & Schneider (2019)	*N* = 90; age: 8–15 years	Income (continuous)	Vocabulary	Resting-state EEG	SES moderated the association between alpha amplitude and vocabulary—positive in lower SES only	Buffering
Noble et al. (2006)	*N* = 38; age: 6–9 years	Composite SES (education, occupation, income-to-needs; continuous)	Nonword reading	fMRI	SES moderated the relation between phonological awareness and left fusiform activation and attenuated the association in higher SES	Buffering
Ozernov-Palchik et al. (2019)	*N* = 129; age: 58–80 months	Parental education (continuous)	Reading outcome	DTI	SES moderated the relation between FA in ILF (T1) and reading (T2)—effect found only in lower SES	Buffering
Ursache et al. (2016)	*N* = 1,082; age: 3–20 years	Parental education and income (continuous)	Cognitive flexibility	DTI	SES moderated Flexibility × White Matter Volume/Integrity relations (cingulum, SLF)—negative in lower SES	Buffering
Demir et al. (2015)	*N* = 40; age: 9–12 years	Parental education (continuous)	Mathematical fluency	fMRI	SES moderated Math Fluency × Brain Activity relations: high SES → verbal (left MTG) and low SES → spatial (right IPS)	Verbal scaffolding
Demir-Lira et al. (2016)	*N* = 33; age: 8–16 years	Hollingshead Index (continuous)	Mathematical fluency (change)	fMRI	SES-patterned brain activity at T1 predicted math improvement over 3 years	Verbal scaffolding
Demir-Lira et al. (2021)	*N* = 39; age: 9–14 years	Caregiver education (continuous)	Deductive reasoning	fMRI	For set-inclusion problems, SES moderated the relation between parietal (SPL/precuneus) activity and nonverbal skills—positive in low SES and negative in high SES; for linear-order problems, SES moderated the relation between RMFG activity and verbal skills, with a positive relation in the lower SES group but not in the highest	Verbal scaffolding
Gullick et al. (2016)	*N* = 42; age: 7–13 years	Parental education (continuous)	Word reading ability	DTI	SES moderated Reading × FA relations across tracts; results were complex, but as the authors reported, higher SES children showed a positive relationship between fractional anisotropy and reading skill in left-hemisphere tract clusters, whereas lower SES children showed a positive relationship in the right-hemisphere homologues	Verbal scaffolding
D’Angiulli, Van Roon, et al. (2012)	*N* = 28; age: 11–15 years	Hollingshead Index (dichotomous)	Selective auditory attention	EEG/ERP	SES moderated Theta Power × Attention relation; low SES → higher theta ignoring tones and high SES → the opposite	Adaptation: The authors proposed that vigilance is adaptive in unpredictable contexts
Ellwood-Lowe et al. (2021)	*N* = 6,839; age: 9–10 years	Poverty status (income-to-needs; dichotomous)	Inhibitory control, flexibility, and reasoning	Resting-state fMRI	SES moderated LFPN-DMN Connectivity × Performance relation; stronger connectivity was beneficial in low SES	Adaptation: The authors proposed that DMN engagement may aid self-monitoring and creative mind-wandering under adverse contexts
Stevens et al. (2009)	*N* = 32; age: 3–8 years	Maternal education (dichotomous)	Selective auditory attention	ERP	SES moderated ERP differences (attended vs. unattended)—smaller effects in low SES	Adaptation: The authors proposed that broad attention is adaptive amid threats
Wray et al. (2017, Year 1)	*N* = 47; age: 3–4 years	SES group by maternal education (dichotomous)	Selective auditory attention	ERP	SES × ERP interaction: high SES → larger amplitude for attended probes and low SES → no difference	Adaptation: The authors proposed that reduced suppression may reflect adaptive vigilance in chaotic environments

Note: Studies are organized by the proposed mechanism of qualitative SES difference (buffering, verbal scaffolding, and adaptation). For adaptation studies, the presumed relevance of SES to the neurocognitive process is summarized. SES = socioeconomic status; EEG = electroencephalography; ERP = event-related potential; fMRI = functional MRI; LFPN = lateral frontoparietal network; DMN = default mode network; MFG = middle frontal gyrus; IFG = inferior frontal gyrus; SFG = superior frontal gyrus; CT = cortical thickness; RMFG = rostral middle frontal gyrus; DTI = diffusion tensor imaging; FA = fractional anisotropy; MTG = middle temporal gyrus; IPS = intraparietal sulcus; SPL = superior parietal lobule; ILF = inferior longitudinal fasciculus; SLF = superior longitudinal fasciculus; IFOF = inferior fronto-occipital fasciculus. T1 = Timepoint 1; T2 = Timepoint 2.

It should be noted that the studies reviewed here serve only as evidence for the existence of neurocognitive processes that differ across levels of SES independent of performance level. We do not know what proportion of studies in the literature might exhibit such a moderation relation because we lack information about studies that did not test for an interaction or tested for an interaction but did not find one and did not report this. Nevertheless, the studies reported here did test the statistical significance of SES moderation and, of 19 articles that reported the appropriate tests, 15 reported evidence of moderation. This could be viewed as a small collection of existence proofs showing that SES disparities in cognition do not invariably indicate a simple quantitative difference, with lower SES children deficient in the same neurocognitive resources used by higher SES children.

### Mathematics

Basic math proficiency is an essential goal of education and shows robust SES disparities at all grade levels (for a review, see Silver & Libertus, 2022; see also White et al., 2016). The neural substrates of mathematical cognition, which are mentioned in this summary, include multiple regions in the parietal and frontal cortex. These regions are recruited for mathematics but also subserve more general roles, including quantity perception, spatial representation, verbal fact retrieval, working memory, and cognitive control (Dehaene, 2011). Some of the clearest evidence for SES moderation of neurocognitive abilities comes from the domain of mathematical cognition. In two studies, Demir et al. (2015) and Demir-Lira et al. (2016) found that the association between young children’s arithmetic skills and their reliance on verbal versus spatial neural representations varied by SES. In Demir et al. (2015), a localizer task first identified verbal and spatial processing areas, which were then used to analyze brain activity during an arithmetic task. In higher SES children, task performance was related to activity in one of the verbal areas, the left middle temporal gyrus, but not in the spatial area, the right intraparietal sulcus. In lower SES children, these relations were reversed, and task performance was related only to the spatial area. This was true independent of the level of performance.

Demir-Lira et al. (2016) then examined SES differences in the neural bases of math skill improvement over time in a 3-year longitudinal study. As before, localizer tasks identified verbal and spatial regions for these students, and SES moderated the relation between math gain and reliance on verbal versus spatial regions. More improvement was observed in higher SES students who relied more on verbal areas (left inferior frontal gyrus) and in lower SES students who relied more on spatial regions (right superior parietal sulcus/precuneus). The authors’ interpretation was that more robust manipulation of preferred representations at baseline allowed children to progress more rapidly.

The results of these two studies (Demir et al., 2015; Demir-Lira et al., 2016) are consistent with two widely supported generalizations concerning language and child development. First, many aspects of linguistic ability are positively correlated with SES across developmental stages (Hoff, 2013; Pace et al., 2017). Second, language plays an important role in the development of mathematical skills (LeFevre et al., 2010). The SES disparity in childhood mathematical cognition has been related to disparities in verbal ability (Duncan & Magnuson, 2005; Galindo & Sonnenschein, 2015; Jordan et al., 1992). Together, these findings suggest that this relation is only part of the story. Language disparity not only contributes to quantitatively different levels of mathematical achievement but also may actually result in qualitative differences in neurocognitive processes underlying that achievement.

### Language

Like math, language skills are crucial for academic success and show persistent SES disparities (Hoff, 2013; Pace et al., 2017). Are these disparities invariably quantitative, with higher and lower SES children carrying out the same linguistic processes with different degrees of success, or might SES correlate with how, qualitatively, language is processed?

The neural substrates of language, summarized here as background for the functional MRI (fMRI) studies that follow, include a set of mostly left-lateralized regions spanning the frontal and temporal lobes. In contrast to traditional “modular” interpretations of the brain bases of language, these areas are now viewed as functioning together as an integrated whole, with more clear-cut boundaries between the language system proper and more general-purpose perceptual and motor systems (Fedorenko et al., 2024). To take reading as an example, visual orthographic patterns are translated into phonology and thence to semantics. The need to coordinate vision, phonology, and semantics suggests that connectivity will be essential for reading, and indeed several white matter tracts play an important role. These include the arcuate fasciculus, the superior longitudinal fasciculus (SLF), and the inferior longitudinal fasciculus (ILF).

The search identified seven studies of language, six of which found qualitative differences. Two studies of reading focused on white matter tracts in relation to reading in elementary school children. Ozernov-Palchik et al. (2019) examined the three left-hemisphere tracts just mentioned and their right-hemisphere homologues in prereaders and tested their relation to reading ability in second grade. The integrity of the bilateral ILF was found to predict later reading performance. Of interest here, the ILF-reading relation was moderated by SES such that it predicted performance for lower SES but not higher SES children. This pattern suggests a buffering effect of a higher SES upbringing (as initially proposed by Noble et al., 2006; for details of the effect, see the Discussion section) so that those who begin at an anatomical disadvantage can nevertheless attain good reading ability with the support of abundant home language and literacy experiences and effective schools.

Gullick et al. (2016) also investigated the role of white matter in reading, adopting a whole-brain approach rather than focusing on predefined tracts. They found interactions between SES and reading skill in the bilateral temporal ILF and the left SLF. Subgroup analyses showed positive relations between fractional anisotropy (FA) and reading skill in regions in the right hemisphere for lower SES but in regions of the left hemisphere for higher SES, including the left ILF. This differs from Ozernov-Palchik et al. (2019), who did not find significant relation between FA and reading skill in the left ILF for higher SES children. The inconsistency may be due to design and sample differences. Ozernov-Palchik et al. (2019) examined connectivity in prereaders and measured reading ability 2 years later, whereas Gullick et al. (2016) focused on the concurrent brain-behavior relationship in readers. Additionally, there were important sample differences: The sample in Ozernov-Palchik et al. (2019) showed the expected relation of SES to reading ability, but the older elementary and middle school students in Gullick et al. (2016) did not.

An early fMRI study of reading by Noble et al. (2006) examined the relation of phonological awareness (PA) to activation of various reading-related regions during a nonword perception task. Activity in the visual wordform area, located in the left fusiform cortex, was positively related to PA as expected. However, SES moderated this relationship, which was pronounced in the lower SES children and attenuated in the higher SES children. The authors proposed that higher SES environments provide more abundant exposure to language and literacy, which supports orthographic processing in children with weaker PA. In other words, weakness in PA, which normally impairs reading, can be buffered by exposure to a richer array of experience with the visual, phonological, and semantic aspects of words.

Brito et al. (2017) addressed both reading ability and vocabulary in relation to a broad structural measure: overall cortical thickness. For both abilities, thinner cortex was associated with better performance, and significantly more strongly for children from lower SES homes. This is again consistent with the idea that more linguistically enriched environments of higher SES children reduce their dependence on anatomical constraints.

Conant et al. (2017) measured individual differences in the categorical perception of phonemes, an essential ability for understanding spoken language. fMRI showed SES differences in the neural correlates of categorical perception. Although the level of performance was related to activation within the left inferior frontal gyrus for all children, those with poor categorical perception showed differences in the activation of other left prefrontal areas, and this was particularly true in the case of higher SES children. The authors did not offer a confident interpretation of this finding but suggested that higher SES may allow children to recruit additional resources, specifically within the left prefrontal cortex, when performing a phoneme discrimination task.

Maguire and Schneider (2019) assessed resting-state EEG in relation to single tests of vocabulary and working memory performance, the latter of which we discuss in the Executive Function section. Vocabulary was positively related to SES, and SES interacted with alpha power to predict vocabulary (a positive relation between alpha power and vocabulary for lower SES children). The authors noted the similarity of this pattern to Brito et al.’s (2017) finding of stronger brain-behavior relations for children from low-SES homes.

In contrast, Decker et al. (2020) measured the relation between anterior and posterior hippocampus volumes and vocabulary size (and memory, as discussed below). Although anterior hippocampus volume predicted vocabulary size for all participants, SES did not moderate the strength of this relation.

To summarize, of the seven published studies examining neural and behavioral measures of reading, vocabulary, and spoken-language perception in higher and lower SES children, all but one reported qualitative differences. Many of these differences are consistent with environmental buffering, whereby children who are neurally at risk for poor language skills benefit from supporting knowledge and skills available in language-rich, higher SES environments.

### Reasoning

Reasoning is the ability to make inferences and draw novel conclusions from given information (Goel & Dolan, 2004) and can take the form of inductive or deductive inference. Inductive inference involves abstracting a generalization from multiple given specific examples, and deductive inference involves the use of general premises to reach a specific conclusion. Both forms of reasoning ability are predictive of academic achievement (Gómez-Veiga et al., 2018; Green et al., 2017). Like the other examples of complex cognitive processes required for school success, reasoning engages networks of prefrontal and parietal regions in both hemispheres, including those used for spatial cognition and those used for language.

Deductive reasoning is itself a varied category that includes set-inclusion problems (e.g., “All tulips are flowers. All flowers are plants. Does this imply that all tulips are plants?”) and linear-order problems (e.g., “Tom is taller than Bill; Bill is taller than John. Does this imply that Tom is taller than John?”). Set-inclusion problems are typically, but not invariably, solved with language, whereas linear-order problems are more likely to be solved using spatial representations (Polk & Newell, 1995; Trabasso et al., 1975).

Demir-Lira et al. (2021) examined the neural basis of deductive reasoning in children in relation to their family SES. Despite the absence of an SES effect on deductive reasoning accuracy, there was nevertheless an effect of SES on brain activity. Set-inclusion problems elicited more activity in language areas for higher SES children than for lower SES children. Instead, lower SES children with strong nonverbal skills showed more parietal visual-spatial activity compared with higher SES children with similarly strong nonverbal skills. Consistent with Demir-Lira et al.’s findings concerning mathematical performance and reading (Demir et al., 2015; Gullick et al., 2016), this study suggests that lower compared with higher SES children may also rely more on spatial regions for reasoning about set-inclusion problems.

Inductive reasoning has been studied in higher and lower SES children using the matrix reasoning task (Raven, 1965). Leonard et al. (2019) studied the relations between inductive reasoning, SES, and cortical thickness. Scores on this type of test are typically correlated with structures of the frontoparietal network. Although matrix reasoning in this study was correlated as expected with the rostral lateral prefrontal cortex (Bunge et al., 2009), the relation held only for the lower SES children. This same general pattern was observed with a smaller adolescent sample without a statistically significant result for moderation. Nevertheless, similar to the child sample, for low-SES individuals the relation of performance to thickness was significant, and for high-SES individuals it was not.

Another study reported SES moderation effects on matrix reasoning ability and its relation to functional brain connectivity at rest (Ellwood-Lowe et al., 2021). The lateral frontoparietal network (LFPN) and the default mode network (DMN) are generally negatively correlated because the LFPN is thought to turn down activity in the DMN when it is engaged in task performance or attention to information in the world rather than in the mind. The strength of this negative correlation predicts children’s test scores (Sherman et al., 2014). However, Ellwood-Lowe et al. discovered that the sign of this relation was negative only for children living above the poverty line. For children living near or below the poverty line, the opposite was true: Better test performance was related to stronger connectivity between the LFPN and the DMN.

Given that there were just three studies of reasoning that met the search criteria, one of deductive reasoning and two of inductive reasoning, it is difficult to generalize about the neural bases of reasoning across levels of SES. The findings of Leonard et al. (2019) fit the general rule of stronger relations between brain measures and performance in lower levels of SES, but neither of the other two lend themselves to straightforward interpretation.

### Attention

The ability to concentrate on a particular input while simultaneously ignoring distractions is known as selective attention and is essential for learning and academic performance. Event-related potentials (ERPs) have been used to study selective attention and have shown that stimuli occurring at the attended location in space show enhanced processing relative to those occurring at unattended locations (Luck & Kappenman, 2011). Localization to particular brain regions is difficult with ERPs, but the timing and amplitude of responses is used to make inferences regarding brain activity. Stevens et al. (2009) instructed 3- to 8-year-old children to attend to a recorded story being presented to one ear while a different story was simultaneously presented to the other and to answer questions about the stories afterward. Whereas the ERPs of higher SES children showed the expected attention effect during listening, with attenuated ERPs to stimuli on the unattended side, the ERPs of lower SES children did not differ between the sides. Specifically, ERPs to the attended ear were similar for both groups of children, but for low-SES children the expected attenuation of the response to unattended stimuli was not observed. A later study replicated these findings in a group of 4-year-olds but found equivalent effects of attention on ERPs in 5-year-olds (Wray et al., 2017). In these ERP studies of selective attention, performance was equivalent for higher and lower SES children, and so the interaction tested was between SES, trial type (attended vs. unattended), and brain activity.

D’Angiulli, Van Roon, et al. (2012) used a different auditory selective attention task involving monitoring for a target tone in a series of tones differing in frequency and duration. Again, performance did not differ between lower and higher SES adolescents but showed a trend that was similar to Stevens et al.’s (2009) ERP findings, with the ERP differences between attended and unattended tones being more pronounced for higher SES participants. Unlike in Stevens et al., the significance of the interaction between SES group and attention condition regarding ERP was not reported. However, in the EEG power analysis, frontal theta band power showed opposite patterns in relation to attention condition between SES groups, and this interaction was significant. Higher SES children showed higher theta power for attended than unattended conditions, whereas lower SES showed lower theta power for attended stimuli.

It should be noted that these three studies differ from the others reviewed here in that task performance was not examined as part of the moderation analyses but was instead matched between higher and lower SES groups. In these studies, an SES × Task Condition interaction (e.g., attended vs. unattended channel) predicted neural responses, suggesting that the neural correlates of attentional filtering differ by SES despite equivalent task performance. Specifically, when told to attend to a story in one ear or monitor sounds of a particular pitch, lower SES children showed electrophysiological signs of more complete processing of the unattended stimuli, suggesting that this less focal attentional processing may be adaptive for individuals of lower SES, who spend time in unpredictable and threatening environments (D’Angiulli, Van Roon, et al., 2012).

### Executive function

EF is a set of cognitive processes that work in concert to enable flexible, goal-oriented responding. These processes include working memory, inhibition, and cognitive flexibility, which are related to different regions of the prefrontal cortex as well as other brain areas, including the parietal cortex. EF has been linked to academic achievement (Best et al., 2011; Lawson & Farah, 2017; Titz & Karbach, 2014).

Brito et al. (2017) examined the association between cortical thickness and EF with tests of working memory, inhibition, and cognitive flexibility. They found that a relatively thinner cortex was associated with better performance in inhibition and cognitive flexibility tasks, and this relation was significantly stronger in children from lower income families. This is consistent with a buffering effect of higher SES on the neural risk factor of a thicker cortex. In contrast, the moderation result regarding working memory was not significant. Using the same data set, Ursache et al. (2016) examined the relation between EF and white matter integrity (as measured by diffusion tensor imaging) and volume across the whole brain and in tracts important for EF. Lower SES children showed a positive association between cognitive flexibility and white matter integrity and volume across the brain and in two of the tracts of interest (the cingulum bundle and superior longitudinal fasciculus), but these associations were nonsignificant or negative in higher SES children. These results suggest possible buffering effects of higher SES. However, the statistical moderation analyses on inhibition and working memory were not significant.

In addition to relating performance on an inductive reasoning task to network connectivity in higher versus lower SES children, Ellwood-Lowe et al. (2021) also examined performance on two EF tasks: testing cognitive flexibility and inhibitory control. Although SES did not moderate the relation of LPFN–DMN connectivity to inhibitory task performance, it did moderate the relation for the cognitive flexibility task.

Three studies did not find evidence for SES moderation of EF–brain activation relations. In Maguire and Schneider (2019), neither SES nor the SES by resting-state EEG interaction predicted working memory as measured by a digit span task. Cascio et al. (2022) used a go/no-go task to measure inhibitory control in adolescents and assessed the association between SES, task performance, and fMRI brain activation. Higher SES was associated with a greater brain response in the classic response inhibition network (Aron et al., 2014), but SES was not related to task performance, nor did it moderate the relation between performance and brain activation. Also using a go/no-go task, St. John et al. (2019) reported no significant interaction between SES and trial type (go vs. no go) when predicting the P3b ERP amplitudes that indexed inhibitory control and attention during the task.

The relations between neural measures, SES, and EF were heterogeneous in this review. This may in part be due to different ways of subdividing EF (see Miyake et al., 2000) and changes to its factor structure with development (Laureys et al., 2022). The strength of the relation between SES and EF may also vary depending on the specific EF subcomponent (Lawson et al., 2018). Consequently, the question of why SES moderation has been observed in certain aspects of EF but not in others remains an open one.

### Memory

The learning of new information (declarative memory) is essential for school success (Hassevoort et al., 2018) and is related to SES (Herrmann & Guadagno, 1997). The hippocampus, a brain region vital to declarative memory (Tulving & Markowitsch, 1998), was studied in relation to both memory ability and SES in two studies of children’s hippocampal volume. Yu et al. (2018) reported an absence of SES moderation of the relation of hippocampal volume to memory ability. Decker et al. (2020) also reported the absence of a statistical interaction between SES, memory ability, and the separately measured volumes of the anterior and posterior hippocampus.

In sum, two studies found that the relation between children’s memory and hippocampal volume was not moderated by SES. Within and across the five domains of neurocognitive ability reviewed here, outcomes were varied. Consistency was limited by the many different tasks and brain measures used. Nevertheless, some trends could be discerned, and these are summarized next.

## Discussion

Many authors of the studies reviewered here offered possible accounts of mechanisms based on the ways in which knowledge and skill acquisition differ in lower and higher SES environments. In the current article, we identified three main types of mechanisms that could account for findings of qualitative SES differences. Three broad categories are described here.

### Mechanisms leading to qualitative SES disparities

#### Buffering

The most frequently hypothesized cause of qualitative differences is the buffering of abilities by the cognitively enriched environment of higher SES homes and schools (Rakesh et al., 2024). This mechanism, proposed by Noble et al. (2006) and subsequently by others, accounts for observed differences in the strength of “classic” or expected brain-behavior relations because higher SES children can draw on their strong language and EF skills, outside of the specific ability being tested, to support their task performance. In phoneme perception, for example, the availability of other associated linguistic knowledge (spoken and written words and sentences) during exposure to phonemes would disambiguate the phonemes and thus facilitate the learning of robust representations. Additionally, EF may support performance in the phoneme discrimination task. Table 1 shows examples of findings on language, reasoning, and EF that can be explained in this way.

#### Verbal scaffolding

A second hypothesized mechanism for qualitative differences is also based on the enriched environments of higher SES children, specifically on linguistic enrichment. Given the robust finding that higher SES children have more exposure to language and consequently better language ability (e.g., Hoff, 2003), they may be more likely to perform ostensibly nonverbal tasks with the help of language. For example, they may “scaffold” their arithmetic calculations with verbal representations, consistent with the findings of Demir et al. (2015). In contrast, the availability of verbal representations to scaffold cognition is more limited for lower SES children, who appear to rely on the other main representational system used in school mathematics, namely spatial representation. Table 1 shows studies of mathematics and reasoning that reveal qualitative differences attributable to differences in verbal scaffolding.

#### Adaptation

The final type of explanation presented here focuses on adaptation to the different demands placed on children’s cognition in lower and higher SES environments. Adaptation accounts of SES disparities have been proposed by several authors (e.g., D’Angiulli, Van Roon, et al., 2012; DeJoseph et al., 2024; Ellwood-Lowe et al., 2021; Leonard et al., 2019; Noble et al., 2021; Taylor et al., 2023). As these authors pointed out, successful adaptations to one environment may be unsuccessful in another. For example, the ability to focus attention and ignore events outside of that focus is an asset in most school and work environments. However, in unpredictable or dangerous environments, in which lower SES children are more likely to find themselves, maintaining broad attentional awareness is adaptive. Adaptation accounts are powerful, possibly too powerful in their flexibility to provide explanations for a wide range of findings, which can make them subject to post hoc reasoning. Ideally, the accounts of lower and higher SES adaptations would have some degree of independent plausibility, as in the utility of broadly allocated spatial attention in dangerous neighborhoods.

### Broader implications, from social justice to classroom pedagogy

#### Understanding versus blaming

Starting in the 1970s, education researchers argued that socioeconomic, racial, and ethnic disparities in school achievement might better be characterized as “differences” between higher and lower performing groups rather than as the more value-laden “deficits” afflicting the lower performing group (Williams, 1970). The difference/deficit distinction is similar, in some ways, to the qualitative/quantitative distinction explored here. “Differences,” like qualitative differences, can take the form of different cognitive strengths, with the strengths of one group better matching methods of instruction and assessment than the strengths of the other. “Deficits,” like quantitative differences, refer to insufficient levels of the same neurocognitive resources used by all individuals. Yet the distinction presented here, between qualitative and quantitative differences, diverges from the earlier theorizing in several ways.

First, difference-based accounts of achievement gaps were seen as a moral improvement over deficit-based accounts. As Valencia (1997) wrote, concerning the history of deficit thinking, “the model is rooted in ignorance, classism, racism, sexism, pseudoscience and methodologically flawed research” (p. xii). It has been seen as a form of blaming the victim in which children’s low cognitive and academic performance is attributed to their own “deficits” rather than structural inadequacies in schools and neighborhoods (Valencia, 1997). In contrast, the quantitative/qualitative distinction proposed here is an empirical generalization that does not itself place blame. Second, the causes of the deficits were variously viewed as genetic or cultural, each of which has been found offensive in its own way (Foley, 1997; Valencia, 1997). The quantitative/qualitative distinction, as an empirical generalization, is agnostic about the causes of SES achievement gaps, although causal mechanisms such as the three mentioned earlier are compatible with specific findings of qualitative differences. Third, apparent deficits could be viewed as the result of differences (Valencia, 1997), giving “differences” in-principle applicability to achievement gaps in all academic and cognitive domains. In contrast, we see no evidence that all SES disparities are qualitative. Quantitative differences are a default assumption, supported by findings of neural mediation of SES-cognition relations, except when there is positive evidence of a qualitative difference.

If the foregoing comparison seems to cast the qualitative/quantitative distinction as irrelevant to issues of social justice, silent on the question of causation, and only narrowly applicable, we agree up to a point with this disappointing conclusion. However, useful hypotheses must be specific enough to be falsifiable and have at least some relevant evidence in their favor. On these grounds, evidence of qualitative differences may indeed be relevant to important social and educational questions.

#### Implications for the social-drift versus social-causation issue

A long-standing question in sociology, with implications for policy, is why poverty and low cognitive and academic performance are linked. Two answers have been offered (Murray, 2020). According to the social-drift account, some people are innately less able, intellectually, and this causes them to drift down in SES because of failures at school and work. This has also been called the “social-selection” account. It can explain lower cognitive and academic performance in poor children, who do not influence the SES of their own childhood, by the reasonable assumption that children inherit genetic tendencies toward intellectual capabilities from their parents. According to the social-causation account, the deprivations of poverty cause diminished cognitive and academic ability. These deprivations include everything from prenatal health to the linguistic richness of parent-child interactions. The two views can be summarized as “low ability causes low SES” (drift) and “low SES causes low ability” (causation).

How does the drift/causation distinction relate to the quantitative/qualitative distinction? Social causation could lead to either quantitative or qualitative differences. If the low-SES environment impairs the overall functioning of a cognitive system used by all to carry out a cognitive task, it would result in a quantitative difference. Alternatively, if it impairs just one mode of cognitive functioning (e.g., verbal strategies, focused attention) it will lead to qualitative differences. In contrast, although drift will stratify people on the basis of their capabilities, there is no obvious way that drift would stratify people on the basis of how, qualitatively, they achieve these capabilities, holding the level of capabilities constant. This is analogous to the way natural selection operates on phenotypes, not genotypes.

To the extent that the social-causation account is indicated by the existence of qualitative differences, it bodes well for the feasibility of closing the achievement gap. Granted, it may require enormous resources, but if deprivation is the driving force of low achievement, then relieving deprivation should increase the achievement of low-SES individuals. In contrast, if the social-drift account is correct, then the policy implications are less certain. Genetic effects can be counteracted by experiential interventions so there is neither reason to doubt nor to believe that improving the environments of lower achieving individuals would raise their achievement.

#### Implications for classroom education

What are the broader implications of the qualitative/quantitative distinction for the education of low SES children? Recognizing qualitative differences can reduce the stigma of low SES in academic contexts in much the same way as difference/deficit advocates envisioned. Perhaps more important than stigma is expectancy, which can be self-fulfilling and impact learning if teachers view low-SES children as less likely to perform well in school (Rosenthal & Jacobson, 1968).

More specific implications for the teaching and assessment of children follow from the nature of the qualitative differences inferred from studies such as those reviewed here. It is certainly not surprising to conclude that lower SES children would benefit from more enriched experience, especially linguistic experience, in their early years, and perhaps even with explicit practice with verbal scaffolding.

The existence of qualitative differences in neurocognitive development suggests that methods that are effective for one group may not translate as well to another. An educational program that is successful in teaching higher SES students may not necessarily benefit lower SES students—and vice versa. For example, if lower SES students tend toward more use of spatial thinking, they may not be well served by teaching methods that emphasize verbal thinking in mathematics or reasoning.

### Future directions

The studies reviewed here call attention to the need for inclusive research samples; a narrow range of SES may yield different results depending on what that SES level is (Bornstein et al., 2013). However, inclusiveness alone is not sufficient. Without moderation analyses, aggregate findings may obscure cognitive patterns that differ across SES groups. Additionally, sufficient statistical power is necessary to detect moderation effects. Without a well-powered moderation analysis, findings such as those from Demir et al. (2015) might be misinterpreted as indicating no SES-related differences in the neural substrates of math performance. The erroneous conclusion would be that all students use a combination of verbal and spatial systems rather than primarily verbal or primarily spatial systems depending on their SES.

When SES moderation effects are found, further analyses can explore their mechanisms, for example, by decompositional approaches such as moderated mediation or conditional process models (Hayes, 2018). Attention should also be paid to potential trade-offs between qualitatively different cognitive processes as proposed in the adaptation models (Frankenhuis et al., 2020) and the boundary conditions on when and where they are optimal (e.g., for reliance on spatial representation for math learning, see Demir et al., 2015; for reduced selective attention, see Stevens et al., 2009).

Additionally, although in this article the neural correlates of task performance served as a marker for determining whether common versus distinct cognitive processes were used by children at different levels of SES, the qualitative/quantitative distinction is not necessarily about brain structure or function per se. For example, selective interference experiments (Nedergaard et al., 2023) could indicate SES differences in reliance on verbal versus spatial representations. Similarly, the role of differences in threat sensitivity could be tested by measuring spatial attention allocation in experimentally manipulated environments with high or low threat.

The research discussed so far holds promise for reducing the SES achievement gap and improving the education of low-SES children. Interventions must be tested bearing in mind the possibility that qualitative differences may limit generalizability. In sum, future work must not only refine our understanding of the brain–SES–cognition relationship but also connect that knowledge to real-world contexts, ensuring that insights into underlying mechanisms inform equitable educational practices.

## Transparency

*Action Editor:* Moira R. Dillon

*Editor:* Arturo E. Hernandez

*Author Contributions*

**Lingyan Hu:** Conceptualization; Writing – original draft; Writing – review & editing.

**Martha J. Farah:** Conceptualization; Writing – review & editing.

## References

[bibr1-17456916251409785] Aron A. R. Robbins T. W. Poldrack R. A. (2014). Inhibition and the right inferior frontal cortex: One decade on. Trends in Cognitive Sciences, 18(4), 177–185. 10.1016/j.tics.2013.12.003 24440116

[bibr2-17456916251409785] Best J. R. Miller P. H. Naglieri J. A. (2011). Relations between executive function and academic achievement from ages 5 to 17 in a large, representative national sample. Learning and Individual Differences, 21(4), 327–336. 10.1016/j.lindif.2011.01.007 21845021 PMC3155246

[bibr3-17456916251409785] Boles D. B. (2011). Socioeconomic status, a forgotten variable in lateralization development. Brain and Cognition, 76(1), 52–57. 10.1016/j.bandc.2011.03.002 21458903

[bibr4-17456916251409785] Bornstein M. H. Jager J. Putnick D. L. (2013). Sampling in developmental science: Situations, shortcomings, solutions, and standards. Developmental Review, 33(4), 357–370. 10.1016/j.dr.2013.08.003 25580049 PMC4286359

[bibr5-17456916251409785] Brito N. H. Piccolo L. R. Noble K. G. (2017). Associations between cortical thickness and neurocognitive skills during childhood vary by family socioeconomic factors. Brain and Cognition, 116, 54–62. 10.1016/j.bandc.2017.03.007 28377043 PMC6527098

[bibr6-17456916251409785] Bunge S. A. Helskog E. H. Wendelken C. (2009). Left, but not right, rostrolateral prefrontal cortex meets a stringent test of the relational integration hypothesis. NeuroImage, 46(1), 338–342. 10.1016/j.neuroimage.2009.01.064 19457362 PMC2864011

[bibr7-17456916251409785] Cascio C. N. Lauharatanahirun N. Lawson G. M. Farah M. J. Falk E. B. (2022). Parental education is associated with differential engagement of neural pathways during inhibitory control. Scientific Reports, 12(1), Article 260. 10.1038/s41598-021-04152-4 PMC8741989 34997113

[bibr8-17456916251409785] Conant L. L. Liebenthal E. Desai A. Binder J. R. (2017). The relationship between maternal education and the neural substrates of phoneme perception in children: Interactions between socioeconomic status and proficiency level. Brain and Language, 171, 14–22. 10.1016/j.bandl.2017.03.010 28437659 PMC5602599

[bibr9-17456916251409785] D’Angiulli A. Lipina S. J. Olesinska A. (2012). Explicit and implicit issues in the developmental cognitive neuroscience of social inequality. Frontiers in Human Neuroscience, 6, Article 254. 10.3389/fnhum.2012.00254 PMC3434357 22973216

[bibr10-17456916251409785] D’Angiulli A. Van Roon P. M. Weinberg J. Oberlander T. F. Grunau R. E. Hertzman C. Maggi S. (2012). Frontal EEG/ERP correlates of attentional processes, cortisol and motivational states in adolescents from lower and higher socioeconomic status. Frontiers in Human Neuroscience, 6, Article 306. 10.3389/fnhum.2012.00306 PMC3500742 23181016

[bibr11-17456916251409785] Decker A. L. Duncan K. Finn A. S. Mabbott D. J. (2020). Children’s family income is associated with cognitive function and volume of anterior not posterior hippocampus. Nature Communications, 11(1), Article 4040. 10.1038/s41467-020-17854-6 PMC7423938 32788583

[bibr12-17456916251409785] Dehaene S. (2011). The number sense: How the mind creates mathematics (Rev. and updated ed.. Oxford University Press.

[bibr13-17456916251409785] DeJoseph M. L. Ellwood-Lowe M. E. Miller-Cotto D. Silverman D. Shannon K. A. Reyes G. Rakesh D. Frankenhuis W. E. (2024). The promise and pitfalls of a strength-based approach to child poverty and neurocognitive development: Implications for policy. Developmental Cognitive Neuroscience, 66, Article 101375. 10.1016/j.dcn.2024.101375 PMC11019102 38608359

[bibr14-17456916251409785] Demir Ö. E. Prado J. Booth J. R . (2015). Parental socioeconomic status and the neural basis of arithmetic: Differential relations to verbal and visuo-spatial representations. Developmental Science, 18(5), 799–814. 10.1111/desc.12268 25664675 PMC4522207

[bibr15-17456916251409785] Demir-Lira Ö. E. Prado J. Booth J. R . (2016). Neural correlates of math gains vary depending on parental socioeconomic status (SES). Frontiers in Psychology, 7, Article 892. 10.3389/fpsyg.2016.00892 PMC4911362 27378987

[bibr16-17456916251409785] Demir-Lira Ö. E. Prado J. Booth J. R . (2021). Neurocognitive basis of deductive reasoning in children varies with parental education. Human Brain Mapping, 42(11), 3396–3410. 10.1002/hbm.25441 33978281 PMC8249891

[bibr17-17456916251409785] Dhammi I. K. Kumar S. (2014). Medical subject headings (MeSH) terms. Indian Journal of Orthopaedics, 48(5), 443–444. 10.4103/0019-5413.139827 25298548 PMC4175855

[bibr18-17456916251409785] Duncan G. J. Magnuson K. A. (2005). Can family socioeconomic resources account for racial and ethnic test score gaps? The Future of Children, 15(1), 35–54. 10.1353/foc.2005.0004 16130540

[bibr19-17456916251409785] Ellwood-Lowe M. E. Whitfield-Gabrieli S. Bunge S. A. (2021). Brain network coupling associated with cognitive performance varies as a function of a child’s environment in the ABCD study. Nature Communications, 12(1), Article 7183. 10.1038/s41467-021-27336-y PMC8664837 34893612

[bibr20-17456916251409785] Falk A. Kosse F. Pinger P. Schildberg-Hörisch H. Deckers T. (2021). Socioeconomic status and inequalities in children’s IQ and economic preferences. Journal of Political Economy, 129(9), 2504–2545. 10.1086/714992

[bibr21-17456916251409785] Fedorenko E. Ivanova A. A. Regev T. I. (2024). The language network as a natural kind within the broader landscape of the human brain. Nature Reviews Neuroscience, 25(5), 289–312. 10.1038/s41583-024-00802-4 38609551 PMC13222024

[bibr22-17456916251409785] Finn A. S. Minas J. E. Leonard J. A. Mackey A. P. Salvatore J. Goetz C. West M. R. Gabrieli C. F. O. Gabrieli J. D. E. (2017). Functional brain organization of working memory in adolescents varies in relation to family income and academic achievement. Developmental Science, 20(5), Article e12450. 10.1111/desc.12450 27434857

[bibr23-17456916251409785] Foley D. E. (1997). Deficit thinking models based on culture: The anthropological protest. In Valencia R. R. (Ed.), The evolution of deficit thinking (pp. 113–131). Routledge.

[bibr24-17456916251409785] Frankenhuis W. E. Young E. S. Ellis B. J. (2020). The hidden talents approach: Theoretical and methodological challenges. Trends in Cognitive Sciences, 24(7), 569–581. 10.1016/j.tics.2020.03.007 32360117

[bibr25-17456916251409785] Galindo C. Sonnenschein S. (2015). Decreasing the SES math achievement gap: Initial math proficiency and home learning environments. Contemporary Educational Psychology, 43, 25–38. 10.1016/j.cedpsych.2015.08.003

[bibr26-17456916251409785] Goel V. Dolan R. J. (2004). Differential involvement of left prefrontal cortex in inductive and deductive reasoning. Cognition, 93(3), B109–B121. 10.1016/j.cognition.2004.03.001 15178381

[bibr27-17456916251409785] Gómez-Veiga I. Vila Chaves J. O. Duque G. García Madruga J. A. (2018). A new look to a classic issue: Reasoning and academic achievement at secondary school. Frontiers in Psychology, 9, Article 400. 10.3389/fpsyg.2018.00400 PMC5883086 29643823

[bibr28-17456916251409785] Green C. T. Bunge S. A. Chiongbian V. B. Barrow M. Ferrer E. (2017). Fluid reasoning predicts future mathematical performance among children and adolescents. Journal of Experimental Child Psychology, 157, 125–143. 10.1016/j.jecp.2016.12.005 28152390 PMC5298800

[bibr29-17456916251409785] Gullick M. M. Demir-Lira Ö. E. Booth J. R. (2016). Reading skill–fractional anisotropy relationships in visuospatial tracts diverge depending on socioeconomic status. Developmental Science, 19(4), 673–685. 10.1111/desc.12428 27412229 PMC5995108

[bibr30-17456916251409785] Hair N. L. Hanson J. L. Wolfe B. L. Pollak S. D. (2015). Association of child poverty, brain development, and academic achievement. JAMA Pediatrics, 169(9), 822–829. 10.1001/jamapediatrics.2015.1475 26192216 PMC4687959

[bibr31-17456916251409785] Hassevoort K. M. Khan N. A. Hillman C. H. Kramer A. F. Cohen N. J. (2018). Relational memory is associated with academic achievement in preadolescent children. Trends in Neuroscience and Education, 13, 8–16. 10.1016/j.tine.2018.09.001

[bibr32-17456916251409785] Hayes A. F. (2018). Introduction to mediation, moderation, and conditional process analysis: A regression-based approach (2nd ed.). Guilford Press.

[bibr33-17456916251409785] Herrmann D. Guadagno M. A. (1997). Memory performance and socio-economic status. Applied Cognitive Psychology, 11(2), 113–120. 10.1002/(SICI)1099-0720(199704)11:2<113::AID-ACP424>3.0.CO;2-F

[bibr34-17456916251409785] Hoff E. (2003). The specificity of environmental influence: Socioeconomic status affects early vocabulary development via maternal speech. Child Development, 74(5), 1368–1378. 10.1111/1467-8624.00612 14552403

[bibr35-17456916251409785] Hoff E. (2013). Interpreting the early language trajectories of children from low-SES and language minority homes: Implications for closing achievement gaps. Developmental Psychology, 49(1), 4–14. 10.1037/a0027238 22329382 PMC4061698

[bibr36-17456916251409785] Jordan N. C. Huttenlocher J. Levine S. C. (1992). Differential calculation abilities in young children from middle- and low-income families. Developmental Psychology, 28(4), 644–653. 10.1037/0012-1649.28.4.644

[bibr37-17456916251409785] Laureys F. De Waelle S. Barendse M. T. Lenoir M. Deconinck F. J. A. (2022). The factor structure of executive function in childhood and adolescence. Intelligence, 90, Article 101600. 10.1016/j.intell.2021.101600

[bibr38-17456916251409785] Lawson G. M. Farah M. J. (2017). Executive function as a mediator between SES and academic achievement throughout childhood. International Journal of Behavioral Development, 41(1), 94–104. 10.1177/0165025415603489 28082756 PMC5222613

[bibr39-17456916251409785] Lawson G. M. Hook C. J. Farah M. J. (2018). A meta-analysis of the relationship between socioeconomic status and executive function performance among children. Developmental Science, 21(2), Article e12529. 10.1111/desc.12529 PMC5821589 28557154

[bibr40-17456916251409785] LeFevre J. Fast L. Skwarchuk S.-L. Smith-Chant B. L. Bisanz J. Kamawar D. Penner-Wilger M. (2010). Pathways to mathematics: Longitudinal predictors of performance. Child Development, 81(6), 1753–1767. 10.1111/j.1467-8624.2010.01508.x 21077862

[bibr41-17456916251409785] Leonard J. A. Romeo R. R. Park A. T. Takada M. E. Robinson S. T. Grotzinger H. Last B. S. Finn A. S. Gabrieli J. D. E. Mackey A. P. (2019). Associations between cortical thickness and reasoning differ by socioeconomic status in development. Developmental Cognitive Neuroscience, 36, Article 100641. 10.1016/j.dcn.2019.100641 PMC6969225 30951970

[bibr42-17456916251409785] Luck S. J. Kappenman E. S. (2011). ERP components and selective attention. In Kappenman E. S. Luck S. J. (Eds.), The Oxford handbook of event-related potential components (pp. 296–328). Oxford University Press. 10.1093/oxfordhb/9780195374148.013.0144

[bibr43-17456916251409785] Lurie L. A. Hagen M. P. McLaughlin K. A. Sheridan M. A. Meltzoff A. N. Rosen M. L. (2021). Mechanisms linking socioeconomic status and academic achievement in early childhood: Cognitive stimulation and language. Cognitive Development, 58, Article 101045. 10.1016/j.cogdev.2021.101045 PMC8112571 33986564

[bibr44-17456916251409785] Maguire M. J. Schneider J. M. (2019). Socioeconomic status related differences in resting state EEG activity correspond to differences in vocabulary and working memory in grade school. Brain and Cognition, 137, Article 103619. 10.1016/j.bandc.2019.103619 31655309

[bibr45-17456916251409785] McDermott C. L. Seidlitz J. Nadig A. Liu S. Clasen L. S. Blumenthal J. D. Reardon P. K. Lalonde F. Greenstein D. Patel R. Chakravarty M. M. Lerch J. P. Raznahan A. (2019). Longitudinally mapping childhood socioeconomic status associations with cortical and subcortical morphology. Journal of Neuroscience, 39(8), 1365–1373. 10.1523/JNEUROSCI.1808-18.2018 30587541 PMC6381251

[bibr46-17456916251409785] Miyake A. Friedman N. P. Emerson M. J. Witzki A. H. Howerter A. Wager T. D. (2000). The unity and diversity of executive functions and their contributions to complex “frontal lobe” tasks: A latent variable analysis. Cognitive Psychology, 41(1), 49–100. 10.1006/cogp.1999.0734 10945922

[bibr47-17456916251409785] Murray C. A. (2020). Human diversity: The biology of gender, race, and class (1st ed.). Twelve.

[bibr48-17456916251409785] Nedergaard J. S. K. Wallentin M. Lupyan G. (2023). Verbal interference paradigms: A systematic review investigating the role of language in cognition. Psychonomic Bulletin & Review, 30(2), 464–488. 10.3758/s13423-022-02144-7 35996045

[bibr49-17456916251409785] Noble K. G. Hart E. R. Sperber J. F. (2021). Socioeconomic disparities and neuroplasticity: Moving toward adaptation, intersectionality, and inclusion. American Psychologist, 76(9), 1486–1495. 10.1037/amp0000934 35266751 PMC9092317

[bibr50-17456916251409785] Noble K. G. Houston S. M. Brito N. H. Bartsch H. Kan E. Kuperman J. M. Akshoomoff N. Amaral D. G. Bloss C. S. Libiger O. Schork N. J. Murray S. S. Casey B. J. Chang L. Ernst T. M. Frazier J. A. Gruen J. R. Kennedy D. N. Van Zijl P. . . . Sowell E. R. (2015). Family income, parental education and brain structure in children and adolescents. Nature Neuroscience, 18(5), 773–778. 10.1038/nn.3983 25821911 PMC4414816

[bibr51-17456916251409785] Noble K. G. Wolmetz M. E. Ochs L. G. Farah M. J. McCandliss B. D. (2006). Brain–behavior relationships in reading acquisition are modulated by socioeconomic factors. Developmental Science, 9(6), 642–654. 10.1111/j.1467-7687.2006.00542.x 17059461

[bibr52-17456916251409785] Ozernov-Palchik O. Norton E. S. Wang Y. Beach S. D. Zuk J. Wolf M. Gabrieli J. D. E. Gaab N. (2019). The relationship between socioeconomic status and white matter microstructure in pre-reading children: A longitudinal investigation. Human Brain Mapping, 40(3), 741–754. 10.1002/hbm.24407 30276914 PMC6628244

[bibr53-17456916251409785] Pace A. Luo R. Hirsh-Pasek K. Golinkoff R. M. (2017). Identifying pathways between socioeconomic status and language development. Annual Review of Linguistics, 3(1), 285–308. 10.1146/annurev-linguistics-011516-034226

[bibr54-17456916251409785] Poeppl T. B. Dimas E. Sakreida K. Kernbach J. M. Markello R. D. Schöffski O. Dagher A. Koellinger P. Nave G. Farah M. J. Mišić B. Bzdok D. (2022). Pattern learning reveals brain asymmetry to be linked to socioeconomic status. Cerebral Cortex Communications, 3(2), Article tgac020. 10.1093/texcom/tgac020 PMC9188625 35702547

[bibr55-17456916251409785] Polk T. A. Newell A. (1995). Deduction as verbal reasoning. Psychological Review, 102(3), 533–566. 10.1037/0033-295x.102.3.533

[bibr56-17456916251409785] Rakesh D. McLaughlin K. A. Sheridan M. Humphreys K. L. Rosen M. L. (2024). Environmental contributions to cognitive development: The role of cognitive stimulation. Developmental Review, 73, Article 101135. 10.1016/j.dr.2024.101135 PMC11741553 39830601

[bibr57-17456916251409785] Rakesh D. Whittle S. (2021). Socioeconomic status and the developing brain – A systematic review of neuroimaging findings in youth. Neuroscience & Biobehavioral Reviews, 130, 379–407. 10.1016/j.neubiorev.2021.08.027 34474050

[bibr58-17456916251409785] Raven J. C. (1965). Advanced progressive matrices sets I and II. H. K. Lewis.

[bibr59-17456916251409785] Reardon S. F. Robinson-Cimpian J. P. Weathers E. S. (2014). Patterns and trends in racial/ethnic and socioeconomic academic achievement gaps. In Ladd H. F. Goertz M. E. (Eds.), Handbook of research in education finance and policy (2nd ed., pp. 491–509). Taylor & Francis.

[bibr60-17456916251409785] Romeo R. R. Christodoulou J. A. Halverson K. K. Murtagh J. Cyr A. B. Schimmel C. Chang P. Hook P. E. Gabrieli J. D. E. (2018). Socioeconomic status and reading disability: Neuroanatomy and plasticity in response to intervention. Cerebral Cortex, 28(7), 2297–2312. 10.1093/cercor/bhx131 28591795 PMC5998958

[bibr61-17456916251409785] Rosenthal R. Jacobson L. (1968). Pygmalion in the classroom. The Urban Review, 3(1), 16–20. 10.1007/BF02322211

[bibr62-17456916251409785] Sherman L. E. Rudie J. D. Pfeifer J. H. Masten C. L. McNealy K. Dapretto M. (2014). Development of the default mode and central executive networks across early adolescence: A longitudinal study. Developmental Cognitive Neuroscience, 10, 148–159. 10.1016/j.dcn.2014.08.002 25282602 PMC4854607

[bibr63-17456916251409785] Shultz M. (2007). Comparing test searches in PubMed and Google Scholar. Journal of the Medical Library Association, 95(4), 442–445. 10.3163/1536-5050.95.4.442 17971893 PMC2000776

[bibr64-17456916251409785] Silver A. M. Libertus M. E. (2022). Environmental influences on mathematics performance in early childhood. Nature Reviews Psychology, 1(7), 407–418. 10.1038/s44159-022-00061-z PMC9624502 36330081

[bibr65-17456916251409785] Sripada C. Angstadt M. Taxali A. Clark D. A. Greathouse T. Rutherford S. Dickens J. R. Shedden K. Gard A. M. Hyde L. W. Weigard A. Heitzeg M. (2021). Brain-wide functional connectivity patterns support general cognitive ability and mediate effects of socioeconomic status in youth. Translational Psychiatry, 11(1), Article 571. 10.1038/s41398-021-01704-0 PMC8575890 34750359

[bibr66-17456916251409785] St. John A. M. Finch K. Tarullo A. R . (2019). Socioeconomic status and neural processing of a go/no-go task in preschoolers: An assessment of the P3b. Developmental Cognitive Neuroscience, 38, Article 100677. 10.1016/j.dcn.2019.100677 PMC6969333 31255904

[bibr67-17456916251409785] Stevens C. Lauinger B. Neville H. (2009). Differences in the neural mechanisms of selective attention in children from different socioeconomic backgrounds: An event-related brain potential study. Developmental Science, 12(4), 634–646. 10.1111/j.1467-7687.2009.00807.x 19635089 PMC2718768

[bibr68-17456916251409785] Taylor E. K. Abdurokhmonova G. Romeo R. R. (2023). Socioeconomic status and reading development: Moving from “deficit” to “adaptation” in neurobiological models of experience-dependent learning. Mind, Brain, and Education, 17(4), 324–333. 10.1111/mbe.12351 PMC10750966 38148924

[bibr69-17456916251409785] Titz C. Karbach J. (2014). Working memory and executive functions: Effects of training on academic achievement. Psychological Research, 78(6), 852–868. 10.1007/s00426-013-0537-1 24389706

[bibr70-17456916251409785] Trabasso T. Riley C. A. Wilson E. G. (1975). The representation of linear order and spatial strategies in reasoning: A developmental study. In Falmagne R. J. (Ed.), Reasoning: Representation and process (pp. 201–229). Psychology Press.

[bibr71-17456916251409785] Tulving E. Markowitsch H. J. (1998). Episodic and declarative memory: Role of the hippocampus. Hippocampus, 8(3), 198–204. 10.1002/(sici)1098-1063(1998)8:3<198::aid-hipo2>3.0.co;2-g 9662134

[bibr72-17456916251409785] Ursache A. Noble K. G. , & the Pediatric Imaging, Neurocognition and Genetics Study. (2016). Socioeconomic status, white matter, and executive function in children. Brain and Behavior, 6(10), Article e00531. 10.1002/brb3.531 PMC5064342 27781144

[bibr73-17456916251409785] Valencia R. R. (Ed.). (1997). The evolution of deficit thinking: Educational thought and practice. Falmer Press.

[bibr74-17456916251409785] Von Stumm S . (2017). Socioeconomic status amplifies the achievement gap throughout compulsory education independent of intelligence. Intelligence, 60, 57–62. 10.1016/j.intell.2016.11.006

[bibr75-17456916251409785] White G. W. Stepney C. T. Hatchimonji D. R. Moceri D. C. Linsky A. V. Reyes-Portillo J. A. Elias M. J. (2016). The increasing impact of socioeconomics and race on standardized academic test scores across elementary, middle, and high school. American Journal of Orthopsychiatry, 86(1), 10–23. 10.1037/ort0000122 26752444

[bibr76-17456916251409785] Williams S. (1970). Preliminaries and prospects. In Williams F. (Ed.), Language and poverty: Perspectives on a theme (pp. 1–11). Elsevier.

[bibr77-17456916251409785] Wray A. Stevens C. Pakulak E. Isbell E. Bell T. Neville H. (2017). Development of selective attention in preschool-age children from lower socioeconomic status backgrounds. Developmental Cognitive Neuroscience, 26, 101–111. 10.1016/j.dcn.2017.06.006 28735165 PMC5703215

[bibr78-17456916251409785] Yu Q. Daugherty A. M. Anderson D. M. Nishimura M. Brush D. Hardwick A. Lacey W. Raz S. Ofen N. (2018). Socioeconomic status and hippocampal volume in children and young adults. Developmental Science, 21(3), Article e12561. 10.1111/desc.12561 PMC5668203 28464381

